# Consortium for violence prevention research, leadership training, and implementation for excellence (CONVERGE): a protocol to train science leaders in gender-based-violence and violence-against-children research for impact

**DOI:** 10.3389/fpubh.2023.1181543

**Published:** 2023-06-29

**Authors:** Kathryn M. Yount, Dawn Comeau, Sarah C. Blake, Jessica Sales, Michael Sacks, Hannah Nicol, Irina Bergenfeld, Ameeta S. Kalokhe, Aryeh D. Stein, Daniel J. Whitaker, Dominic Parrott, Hoang Thi Hai Van

**Affiliations:** ^1^Hubert Department of Global Health, Rollins School of Public Health, Emory University, Atlanta, GA, United States; ^2^Department of Behavioral, Social, and Health Education Sciences, Rollins School of Public Health, Emory University, Atlanta, GA, United States; ^3^Department of Health Policy and Management, Rollins School of Public Health, Emory University, Atlanta, Georgia; ^4^Goizueta Business School, Emory University, Atlanta, GA, United States; ^5^School of Medicine, Emory University, Atlanta, GA, United States; ^6^Georgia State University, Atlanta, GA, United States; ^7^Hanoi Medical University, Hanoi, Vietnam

**Keywords:** gender-based violence, implementation science, leadership, mentorship, research capacity strengthening, Vietnam, violence against children

## Abstract

**Background:**

Gender-based violence (GBV) and violence against children (VAC) are two prevalent and highly interconnected global health challenges, yet data and research capacities to study these forms of violence and to generate evidence-based policies and programs remain limited. To address critical shortages in research capacity in Vietnam and to establish a model for other Low- and Middle-Income Countries (LMICs), we are establishing CONVERGE—the Consortium for Violence Prevention Research, Implementation, and Leadership Training for Excellence.

**Methods:**

Based on a needs assessment with partners in Vietnam, CONVERGE will provide a comprehensive research training program supporting 15 long-term, postdoctoral trainees with multi-disciplinary research training in GBV and VAC. We also will offer in-country trainings and short-courses to 40 short-term mid-career academic trainees and 60 short-term practitioner/stakeholder trainees over 5  years to build productive GBV and VAC academic, scientific, and practitioner networks. The CONVERGE training program has four components: (1) 14 h of virtual/in-person annual mentorship training to prepare research mentors and to create a pipeline of future mentors in Vietnam; (2) a one-month intensive research training for long-term postdoctoral fellows at Emory University; (3) a structured 17-month, in-country mentored research project for long-term trainees that results in a peer-reviewed manuscript and a subsequent grant submission; and, (4) week-long in-country intensive translational trainings on implementation science, advanced topics in leadership, and advanced topics in science dissemination. Opportunities for on-going virtual training and professional networking will be provided for CONVERGE trainees and mentors in Vietnam with other trainees and mentors of D43s focused on injury/violence prevention, D43s housed at Emory, and D43s with other institutions in Southeast Asia. To assess the reach, implementation, fidelity, and effectiveness of these four components, we will implement a rigorous, mixed-methods, multi-level evaluation strategy using process and outcome measures. Findings from the evaluation will be used to refine program components for future trainee and mentor cohorts and to assess long-term program impact.

**Discussion:**

Led by Emory University in the US and Hanoi Medical University in Vietnam, CONVERGE represents leading institutions and experts from around the world, with a goal of providing mentorship opportunities for early-career scientists with an interest in violence prevention.

## Introduction

Gender-based violence (GBV) entails any harmful act perpetrated against a person’s will because of their biological sex or gender presentation (1, 2). Acts of GBV inflict physical, sexual, mental, or economic harm, threats of harm, coercion, or other deprivations of liberty in public or private. Violence against children (VAC) includes all forms of violence against people under age 18 years, whether by parents or other caregivers, peers, romantic partners, or strangers. When directed against girls or boys because of their biological sex or gender presentation, acts of VAC are forms of GBV. Common forms of GBV against women, girls, and trans-gender or gender-non-conforming (TGNC) persons include intimate partner violence (IPV) (3–6) and homicide (7, 8), sexual violence (4–6, 9), harmful practices like child, early, or forced marriage (CEFM) (10), honor crimes (11), and human trafficking (12). GBV and VAC are important to consider jointly because they may share risk factors (13–21), often co-occur in households, are linked over the life course (15, 18, 22), can have common and compounding consequences over the life course, can have inter-generational effects, and can co-occur in adolescence, when vulnerability to violence is heightened. Moreover, GBV and VAC are related to a host of negative outcomes as either a cause or consequence, including mental health outcomes, such as depression or anxiety; physical health outcomes, such as immediate injury and long-term physical health problems such as cancer or diabetes; and behavioral health outcomes, such as substance use or unsafe sexual practices (23–27). Thus, GBV and VAC are “hub” foci, such that capacity building to strengthen related scholarship and public health responses will likely have positive ripple effects well beyond the prevention of violence.

Despite the global burden of GBV and VAC, data and research capacities to study these forms of violence and to generate evidence-based policies and programs remain limited (6). Our objective is to address critical shortages in research capacity in Vietnam, where political will is high to expand capacity for research and programs on GBV and VAC and to become a model for other LMICs. We will train mentors, post-doctoral researchers, and practitioners involved in studying and addressing GBV and VAC. To achieve this objective, we have established CONVERGE—the Consortium for Violence Prevention Research, Implementation, and Leadership Training for Excellence. Collaborating institutions are led by Emory University (United States, US) and Hanoi Medical University (HMU, Vietnam) and include the following partners in Atlanta and Vietnam: Georgia State University; the Division of Violence Prevention, Center for Injury Prevention and Control, Centers for Disease Control and Prevention; Haiphong University of Medicine and Pharmacy (UMP); Hue UMP; Ho Chi Minh City UMP. CONVERGE draws on and expands successful training initiatives between Emory University and institutions of excellence in Ethiopia (D43TW009127), Georgia (D43TW007124), India and Ethiopia (D43TW011404), and Nigeria (D43TW010934) for building research and leadership capacity while tailoring curricula to address the identified needs of universities in Vietnam. Our training initiative has three specific aims:

**Aim 1:** To cultivate a diverse group of mentors for research on GBV and VAC in Vietnam by implementing a formal mentorship program that trains productive researchers to be strong scientific and professional mentors (15 early-career long-term trainees; 40 mid-career short-term trainees). This mentorship program will strengthen CONVERGE mentoring teams and institutional capacity in Vietnam.

**Aim 2a:** Strengthen research capacity and enhance human capabilities for robust GBV and VAC research in Vietnam by providing long-term post-doctoral research training and career opportunities to a diverse group of researchers with exceptional potential (15 early-career long-term trainees).

**Aim 2b:** Strengthen capacity to fast-track the translation of evidence into practice by providing in-country short-courses on implementation science, leadership, and science dissemination that connect scholars and practitioners (15 early-career long-term trainees; 40 mid-career short-term trainees; 60 practitioners).

**Aim 3:** Implement a training evaluation plan that assesses the reach, implementation, fidelity, and effectiveness of our four short- and long-term training program components. We will use these findings iteratively to improve the program in real time, and thereby, to maximize favorable individual and institutional impacts.

Our program aligns with national research priorities of Vietnam (28) and leverages over 12 years of collaboration between Emory University and partners in Vietnam, demonstrating the feasibility of achieving our aims. Our empowerment-based approach (29) to mentorship training will create a pipeline of early- and mid-career researchers who are skilled mentors. Our long-term post-doctoral research training is grounded in successful integrated, interdisciplinary training programs with Emory University and other partners and meets identified needs in Vietnam. Our implementation science, leadership, and science dissemination short courses address identified gender gaps in leadership training (30), build on Emory’s successful leadership programs (D43TW009135), and will accelerate the translation of evidence into practice. Our training plan addresses the priorities of several divisions within the National Institutes of Health (NIH) related to women’s health, child health and human development, mental health, minority health and health disparities, alcohol abuse and alcoholism, drug abuse, and general medicine.

CONVERGE members are energized to meet the demand for violence-prevention research, implementation, and leadership training in Vietnam. Over the program period, we expect to train a critical mass of 115 research mentors, post-doctoral scholars, and practitioners in Vietnam in GBV and VAC research, implementation, and leadership. Through successful strategies tested in our prior training programs, we will promote high trainee retention and strengthen institutional capacity in Vietnam. In doing so, we will prepare a consortium of universities in Vietnam to conduct locally relevant GBV and VAC research, training, and best practice that shapes national research and program priorities into the twenty-first century.

### Burden of GBV and VAC in low- and middle-income countries

Estimated rates GBV and VAC are high worldwide (3, 8, 9, 31), but the burden is disproportionate in LMICs. First, 89% of the world’s 3.19 billion youth 0–24 years live in LMICs (31, 32). Among 2–17-year-olds in 2014, 1.35 billion children in LMICs and 135 million in high income countries (HICs) were exposed to any violence, and rates of any violence were higher in LMICs (78% vs. 60%) (31). Second, some gendered forms of VAC—such as the CEFM of girls—are more common in LMICs (11, 33). Third, exposure to gendered and non-gendered violence in childhood increases the risks of revictimization (34), poly-victimization (35, 36), and perpetration (7, 18, 22, 37–41) in adulthood and inter-generationally (42). Fourth, violence against TGNC persons is understudied in LMICs, but limited data from HICs suggest that child abuse, early stigma and discrimination, and physical or sexual violence may be as or more common in TGNC persons than in *cis*gender persons, whose gender identity matches their assigned sex at birth (43). Fifth, VAC and GBV at all ages have cumulative and intersecting mental, physical, and behavioral sequelae (42, 44), some of which are more common or specific to women, girls, and TGNC persons (45). Such sequelae include poor mental health; suicidal ideation and behavior; depression and posttraumatic stress; eating disorders (46), number of sexual partners; sexually transmitted infections, including HIV; unwanted or unplanned pregnancy and abortion; and poor maternal and child health outcomes (47). Thus, despite limited data on many forms of VAC and GBV, larger numbers of youth and some evidence of higher risk create a heavy burden of VAC, GBV, and their intersecting sequelae in LMICs.

### Common social ecology of GBV and VAC: rationale for early, integrated intervention

**Figure 1:**
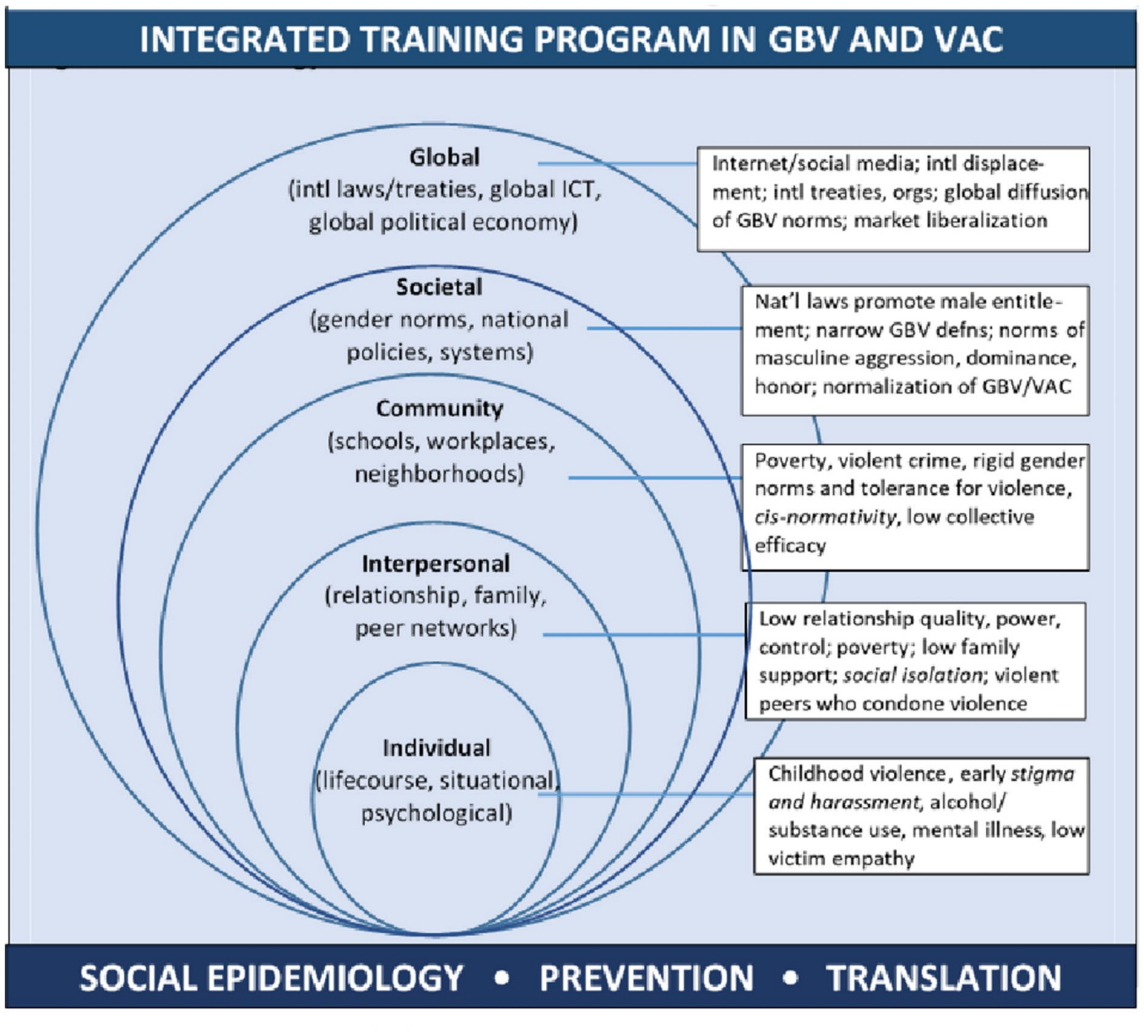
Socio-Ecological Model of Common Risk Factors for Gender-Based Violence and Violence against Children.

According to socio-ecological theories (13, 14, 48), nested inequalities in power and control as well as norms of masculine dominance influence multiple forms of GBV and VAC (Figure 1). This framework supports an integrated approach to study these forms of violence, noting common and *specific* multilevel risk factors for victimization and/or perpetration. Individual-level factors include early exposure to violence, situational factors, such as alcohol or substance use, poor mental health, low victim empathy, and for TGNC persons, early stigma and discrimination (18, 38, 42, 49, 50). Interpersonal factors include low relationship quality characterized by power and control, household poverty and low family support, peer-networks that condone violence, and for TGNC persons, heightened social isolation (38, 42, 51). Community-level factors include living in a socio-economically disadvantaged neighborhood with low collective efficacy, high violent crime, norms accepting of gender inequality and violence (15, 18, 19, 42, 52, 53), and for TGNC persons, cis-normativity. Also important at the community level are factors in schools (54) and workplaces (55), such as organizational tolerance for GBV or VAC, gender and other occupational inequalities, and weak or absent anti-discrimination policies, prevention and response programs, bystander training, and leadership training. Societal or national factors include laws, policies, and systems that privilege *cis*men, that limit opportunities for women and TGNC persons, and that promote narrow definitions of GBV or VAC, rigid gender norms and norms of masculinity tied to dominance and honor, and acceptance of interpersonal violence (21, 42, 56, 57). Global factors include the internet, which may increase access to education and prevention programs as well as exposure to violent sexually explicit material, cyber-bullying, cyber-stalking, and sexual exploitation (58, 59). Other global factors include international displacement, which may elevate the risks of GBV and VAC by weakening community cohesion (60); international treaties and organizations that support the diffusion of anti-GBV and anti-VAC norms and practices (61, 62); and economic liberalization, which may elevate organized crime and, in turn, the diffusion of criminal laws against human trafficking in economically interdependent countries (63). Thus, GBV and VAC share many risk factors across the social ecology (42), and early integrated interventions targeting shared risks may help to prevent both forms of violence.

### Research gaps in LMICs

Recognizing the global burden of GBV and VAC, the 2015 Sustainable Development Goals (SDGs) called on national governments to eliminate all forms of violence against women and girls (SDG5.2), harmful practices such as CEFM (SDG5.3), as well as abuse, exploitation, trafficking, and all forms of violence against children (SDG16.2) (64). This call has prompted a need for valid measures to monitor all forms of GBV (65, 66) and VAC (31) and to understand what works to prevent these forms of violence. Despite increased attention to GBV, research has come largely from HICs, which may or may not apply to LMICs. In LMICs, research on GBV has focused on adult women in urban settings and selected regions (Africa, South Asia), leaving the experiences of adolescent girls, young women, TGNC persons, and entire regions underexplored (36). Studies also focus on certain forms of GBV (e.g., IPV and non-partner sexual violence), lack attention to poly-victimization, use variable, unvalidated measures, and focus prevention programs on single forms of GBV (36, 46, 67). Likewise, despite increasing data collection on VAC, internationally agreed standards of measurement are lacking (68), and the quality of data is uneven. Moreover, reliance on partial definitions of VAC (69) and oversight of hard-to-measure forms (70) likely result in under-estimates of exposure. Attention to social variation in the burdens of VAC or GBV also is limited. Measures to ensure the rigorous implementation of study protocols and to safeguard the protection of participants are lacking (68). As a result, few national research institutes in LMICs have the technical expertise to collect data on GBV and VAC (68).

### Asia Pacific and Vietnam: high prevalence of GBV and VAC but capacity and data gaps

Based on available data from Vietnam (71), lifetime (63%) and prior-year (32%) IPV is common in women 15–64 years. From 2010 to 2019, women’s reports of lifetime physical IPV declined (32–26%), but their reports of lifetime sexual IPV increased (10–13%), especially in women 18–24 years (5–14%). Half of women who report marital IPV never tell anyone, and few (10%) seek formal help. Of women who report physical IPV in marriage, 61% report their child witnessed or overheard the violence, and report behavioral problems in their child (5–12 years) more often than women not reporting physical IPV.

Outside of intimate partnerships, more than 4% of women report child sexual abuse before age 15 years. One in 10 women (11%) report non-partner physical violence since age 15 years, the majority (61%) of whom report a male relative as the perpetrator. Nearly one in 10 women (9%) report non-partner sexual violence since age 15, mostly perpetrated by non-family male acquaintances, co-workers, or strangers. High and increasing rates of some forms of intimate-partner, child, and non-partner violence against women may reflect changing exposure and more openness to discuss sex and sexual violence. Violence against women costs Vietnam an estimated 1.8% of gross domestic product (GDP) and profound individual consequences: 23% who experience physical and/or sexual IPV report physical injuries. Women victims more often than non-victims have a husband who was beaten as a child or whose mother was beaten.

In Vietnam, research on VAC is uncommon. More widely, according to the 2019 Global School-based Student Health Survey in Viet Nam (72), a decline was observed between 2013 and 2019 in the percentage of physical attacks, physical fights, and bullying at school (from 21 to 10% for attacks; from 17 to 8% for fights; and from 23 to 6% for bullying). However, according to the Ministry of Labour, Invalids, and Social Affairs, more than 4,000 children were abused between June 2019 and June 2021 (73). Of these cases, about 90% were girls, more than 66% were children 13–16 years old, and over 293 were children under age 6 years. Based on our research outside Hanoi, 75% of men experience violence in childhood and/or exposure to IPV, and their odds of IPV perpetration (74) and sexually violent behavior (75) increase with such exposure. Across East Asia and the Pacific, the economic loss from DALYS attributable to child maltreatment is an estimated $206 billion in 2012 dollars, or 2% of aggregate GDP (76), and is highest in the Western Pacific region, including Vietnam, at 3% of GDP (76).

Research on TGNC persons in the region is limited, but practices of gender variance, gender pluralism, and non-normative sexualities have long histories in Southeast Asia and Vietnam. These histories are shaped by ethnicity, spirituality, colonialism, HIV-related public-health campaigns, and legal recognition of the rights of gender-variant persons (77). Given the higher lifetime risk of adversity among sexual-minority than non-sexual-minority men (78), and the intersectional stigma experienced by gender-non-conforming, sexual-minority women (79) in parts of Southeast Asia, violence research that jointly considers the intersection of gender and sexuality is needed in Vietnam and the region.

### Needs assessment

Thus, despite the high burdens of GBV and VAC in Vietnam, infrastructure and opportunities for interdisciplinary, integrated research training are nascent. Our history of collaboration in Vietnam and informal needs assessments with HMU revealed several research-training gaps (Table 1). Developing investigator capacity at the post-doctoral level is a critical pipeline to address these needs, and combined with strengthening mentorship capacity, is a powerful strategy toward building institutional capacity for research and programming in violence prevention.

**Table 1:** Needs and challenges researching gender-based violence and violence against children in Vietnam.

Gaps and challenges	How CONVERGE will address these gaps/challenges
Medical and doctoral trainees have few ways to support their work when transitioning from a trainee dependent on a graduate advisor to independence. Limited in-country opportunities for scientific growth during this transition has contributed to a recent “brain drain.”	Fund post-doctoral fellows to gain 1 month of exposure to state-of-the-art research facilities and US research culture Provide 18 months of postdoctoral stipends Support in-country mentored research projects with dedicated research funds ($5,000 per trainee) Provide in-country mentored research projects with competitive matched funding from US universities
Need for training opportunities in social and behavioral sciences (e.g., epidemiology, biostatistics, sample surveys, GBV/VAC measurement, prevention trials, natural experiments, translation research) and qualitative/mixed-methods approaches that address risk and protective factors across the social ecology and in gender-variant groups (Figure 1)	Social and behavioral science research methods and qualitative/mixed-methods research address risk/protective factors across the social ecology and vulnerable groups are integral to the 1-month intensive research training in the US Monthly webinars and annual in-country trainings will offer on-going support to all trainee cohorts on topics relevant to their research
Few opportunities for trainees to engage in the study of GBV and VAC, their interlinkages, and health outcomes, including in locally salient vulnerable populations and gender-variant groups	Program faculty bring expertise in diverse forms of GBV and VAC, research methods (national surveys; sampling methods for hard-to-reach persons; mixed-methods; cohort studies; prevention trials) Program faculty agree to provide access to datasets, other experts
Limited interdisciplinary research and training offering a “socio-ecological” view of GBV and VAC risk and protective factors, their interlinkages, and diverse behavioral, health, social, and economic outcomes; limited access to relevant quantitative and qualitative datasets in diverse samples	Program faculty have diverse methods, content expertise Repeat national VAWG survey (2010/19), multi-country Violence Against Children Surveys and men’s IPV perpetration surveys in the region; intervention cohorts; qualitative VAWG data offer opportunities to study wide-ranging exposures and outcomes in diverse samples
Gaps between researchers, needs, and policy and no formal ways to connect researchers with practitioners (clinicians and health administrators) to translate evidence into practice and policy	Short courses will connect researchers and practitioners in implementation science, advanced topics in leadership, and advanced topics in science dissemination Monthly webinars will be open to long-term trainees and interested practitioners within and beyond CONVERGE Webinars will be recorded and uploaded to websites to expand reach Dissemination workshop (Year 5) will share findings with practitioners and policy stakeholders, increasing potential for research uptake
Shortages of soft skills, like science communication, scientific leadership, and organizational management to support expansion and sustainability of research institutions in LMICs	One-third of the short-course is devoted to soft skills. All trainees will complete workstyle and leadership assessments and a debriefing to enhance self-awareness Webinars and readings will supplement training
To ensure long-term sustainability and expansion of in-country training, no programs assist researchers to become effective mentors.	We designed a 14-h course to improve mentor effectiveness and will deliver this in segments over the course of each 18-month post-doctoral cohort with 40 short-term scholars and all 15 long-term trainees, who then will become in-country mentors

### Feasibility: adapting prior training successes

To strengthen research training in the long term, retaining skilled investigators in-country is essential. Emory University has shown through its other D43 training grants the feasibility of training and retaining research scholars in-country by offering them quality opportunities for research and career development (D43HD065249, D43TW009135, D43TW009127, D43TW007124). The present D43 applies this institutional learning and critical design innovations, such as: a revised mentor-training program that engages in-country mentors over the course of each of three 18-month post-doctoral fellowship training periods; competitive funding for follow-on research projects and research-dissemination activities; foundational and advanced training in leadership that is attentive to cross-cultural issues of diversity, equity, and inclusion; the seamless integration of graduates from our long-term post-doctoral training program into an expanding network of CONVERGE mentors; co-hosting of trainee and mentor training opportunities that facilitate cross-cultural learning and networking across D43 training programs at Emory, in Southeast Asia, and focused on violence prevention; and a comprehensive and rigorous plan to evaluate the reach, fidelity, effectiveness, and public-health impact of the program. Together, these innovations are designed to develop a cadre of early-career scientific leaders; an expanded network of scientific mentors; multi-institutional scientific capacity; and translational public-health impact in Vietnam.

## Methods/Design

### Converge global training program and program evaluation

#### Administrative structure

**Figure 2:**
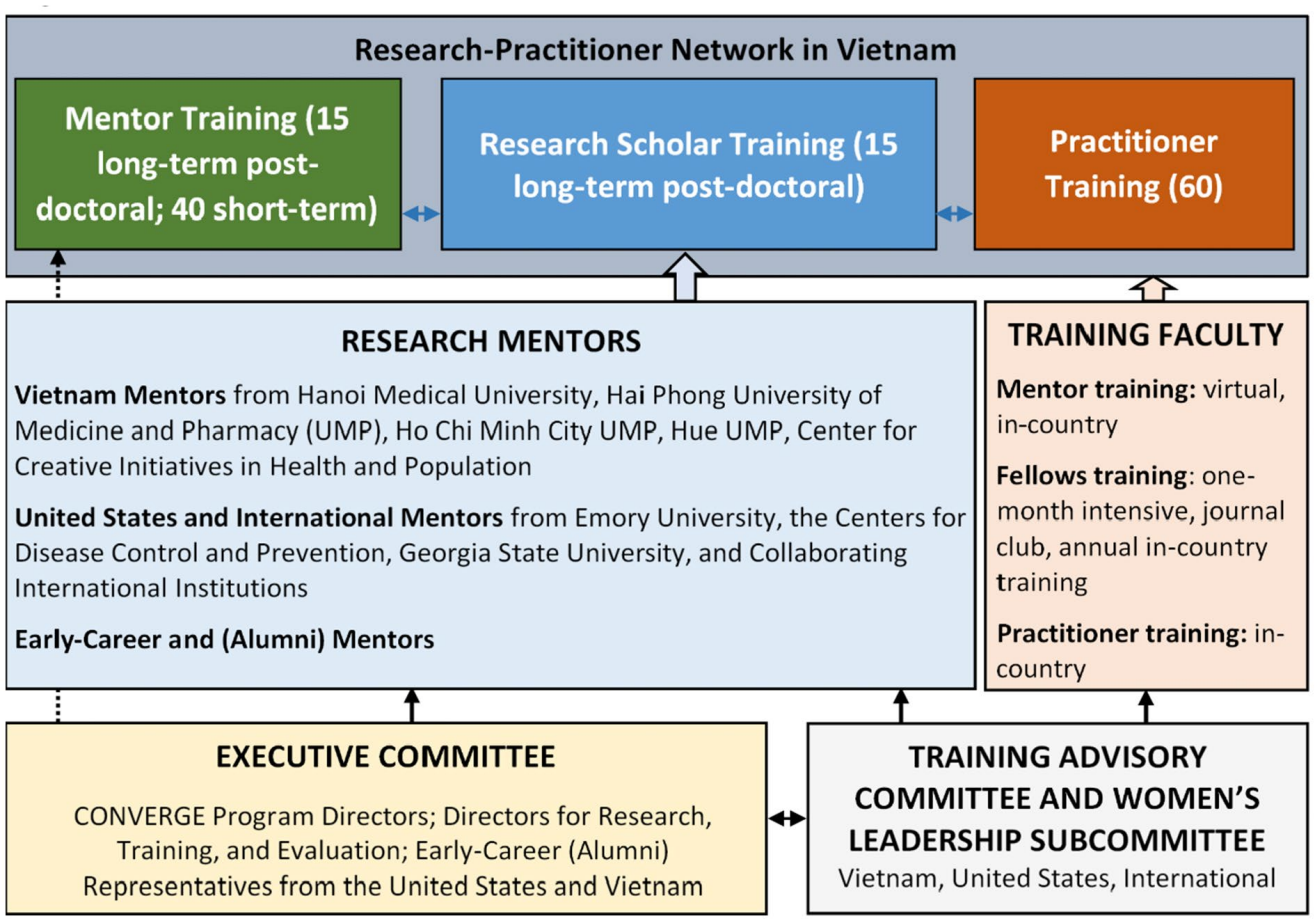
CONVERGE administrative structure.

CONVERGE is a collaboration of partners with expertise in GBV and/or VAC at universities and scientific agencies in the US, Vietnam, and internationally. The primary institution in the US is Emory University, and US partner institutions include Georgia State University and the Division of Violence Prevention, Center for Injury Control and Prevention, Centers for Disease Control and Prevention (CDC). In Vietnam, the primary partner is Hanoi Medical University (HMU), and consortium partners include Haiphong University of Medicine and Pharmacy (UMP), Ho Chi Minh UMP, and Hue UMP. Other international partners are based in Europe and South Africa (Figure 2).

CONVERGE has assembled a world-class, diverse, and interdisciplinary Executive Committee and Program Faculty (Figure 2). The leadership team brings multi-disciplinary and complementary expertise, and extends a successful collaboration since 2010 between the PI at Emory University and institutions in Vietnam, which has expanded opportunities for early-career and senior investigators. The Executive Committee includes Program Directors providing programmatic leadership and oversight following a Multi-Program Director Leadership Plan. The PI and Program Director at Emory ensures adherence to program vision, grant management, financial accountability, and reporting to NIH. The MPI and co-Director at HMU oversees the recruitment of all long-term and short-term trainees, identification of appropriate in-country research mentors, in-country mentored research training, coordination of partner universities in Vietnam, and annual in-country trainings with long-term trainees, mentors, and practitioners. The Director for Research oversees the review and selection of long-term post-doctoral applicants, the matching of selected trainees with primary US mentors and mentoring teams, and research progress of each long-term trainee. The Director for Training oversees the one-month post-doctoral training intensive at Emory, mentorship training, and monthly journal-club webinars for long-term trainees. The Director of Evaluation oversees a comprehensive, mixed-methods evaluation plan for the entire training program. A collaborating investigator at HMU partners on all training and research activities, and early-career representatives from the US and Vietnam participate in programming and have opportunities for career-development on the CONVERGE Executive Committee.

To support this leadership team, a Training Advisory Committee (TAC) includes members from Atlanta-based institutions, Vietnam-based institutions, and international institutions. The CONVERGE TAC includes global experts in research on women’s health, GBV, VAC, and strategies to prevent injury and violence in LMICs. Members of the TAC were selected for their experience as current Directors of NIH-funded D43 training grants and/or Centers for research and training in the U.S., Vietnam, and globally. A women’s leadership sub-committee of the TAC monitors the selection and progress of women trainees to ensure the successful completion of their training program and opportunities for leadership development.

#### Program overview

CONVERGE provides a comprehensive research training program to support 15 long-term, postdoctoral trainees with multi-disciplinary research training in GBV and VAC. We also offer in-country trainings and short-courses to 40 short-term, mid-career academic trainees and 60 short-term practitioner/stakeholder trainees over 5 years (*n* = 115) to build productive GBV and VAC academic, scientific, and practitioner networks in Vietnam. Training components are based on the needs assessment conducted with partner institutions in Vietnam.

The CONVERGE research training program has four components: (1) an annual three-day mentorship training to prepare *research mentors* to build local mentoring capacity, effective and sustainable mentoring relationships, and a pipeline of future mentors in GBV and VAC in Vietnam; (2) a one-month multi-disciplinary intensive research training at Emory University that provides *long-term trainees* opportunities to acquire skills and expertise in rigorous, state-of-the-art, and reproducible research methodologies in GBV and VAC research; (3) a structured 17-month, in-country mentored research project for *long-term trainees* to apply state-of-the-art methods in GBV and VAC research to their project that results in a peer-reviewed manuscript and a grant submission to support the next phase of their research careers; and (4) a week-long in-country intensive translational training on implementation science, leadership, and research dissemination for long-term and short-term trainees to strengthen capacity and build networks between science and practice communities. These training initiatives will increase the number of scientists and health professionals who are able to implement rigorous GBV and VAC research and evidence-based programming at their home institutions as well as throughout national and global GBV and VAC research and professional networks. Based on our needs assessment, the CONVERGE training program is unique and offers training opportunities not yet available from in-country institutions or other externally funded training programs at our partner institutions. Moreover, training is developmentally appropriate for post-doctoral researchers, with the anticipation of building impactful careers as scientific investigators.

Below, program components are reviewed sequentially in their expected order of implementation: (1) selection and training of long-term post-doctoral trainees, (2) training of in-country research mentors, and (3) translational trainings and program evaluation.

### Phase 1: long-term postdoctoral training eligibility, recruitment, and selection

#### Phase 1a: eligibility

A core aim of our training program is to build research capacity and enhance human capabilities for robust GBV and VAC research in Vietnam by providing long-term post-doctoral research training and professional opportunities to a diverse group of early-career researchers. Over the five-year program, we aim to support 15 post-doctoral research scholars across three cohorts. Eligible long-term trainees include early-career faculty, post-doctoral fellows, or PhD-level scientists at a partner institution in Vietnam who seek to obtain didactic and experiential, mentored training foundational for a research career in GBV or VAC. Long-term trainees are selected with attention to: (a) academic and research-related qualifications, (b) quality of the match with institutional and mentored support; (c) commitment to research on GBV or VAC; (d) inclusion of under-represented scientists (e.g., on gender, ethnicity); (e) disciplinary balance; and (f) balanced recruitment to create “clusters of capacity” across partner institutions. Candidate qualifications include a minimum of MD and/or PhD degree or equivalent, passion and commitment to research on GBV or VAC, quantitative or qualitative and critical analysis skills, aptitude for interdisciplinary and collaborative work, and commitment to reduce violence among vulnerable populations in Vietnam. When possible, trainees are selected to balance research interests that span the areas identified as gaps in Vietnam and within Vietnamese institutions (Table 1).

#### Phase 1b: recruitment

The team’s in-country MPI/co-Program Director oversees the recruitment of all trainees, with attention to the aforementioned criteria (Phase 1a). Each year, opportunities for long-term post-doctoral-level training are advertised widely through in-country advertisements, websites, listservs, word of mouth, and the extensive networks of the MPIs, in-country mentors, and current long-term trainees at partner institutions in Vietnam. Attention is given first to potential candidates at partner institutions, to strengthen individual and institutional research capacity with our core partners. Candidates from other institutions who have the appropriate institutional support and commitment to the field also are considered. Advertisements target candidates who are committed to continue GBV- and VAC-related topics in Vietnam. To expedite the recruitment and selection of the first cohort of trainees, partner institutions identified potential individuals at their institutions at the application stage of this training program.

#### Phase 1c: application and selection process

All cohorts of long-term trainee candidates are selected competitively using an application process adapted from existing Emory D43 training programs. Applications are completed in English, the language of most international biomedical, social science, and public health journals. All applications require a: (1) Curriculum Vita, (2) responses to a short demographic and informational survey, (3) letter of support from the applicant’s home institution, (4) letter of recommendation from a local research mentor, and (5) short statement of interest and one-page research proposal. Letters of recommendation should come from faculty in a graduate education program who are current or former supervisors and potential program mentors and should describe why the applicant is a strong candidate for the CONVERGE program. The institutional letter of support should describe the quality and relevance of research conducted at the institution, express commitments to support the trainee throughout the program, and outline a strategy to support the trainee’s integration into a research position upon completion of the program. The statement of interest and research proposal should describe their interest in GBV/VAC research, outline a hypothesis-driven research question on GBV/VAC they wish to pursue during the training period, and describe the data they plan to collect and analyze, timeline, and a statement of the study’s feasibility. Because written and oral proficiency in English is necessary for this training program, all finalists interview with the review committee and respond to questions about their training and research proposal.

The CONVERGE Director for Research (DR) facilitates the selection process for each long-term trainee cohort. For each admission cycle, the DR invites reviewers from the CONVERGE Executive Committee or the extensive pool of program faculty. Reviewers screen each application. Applications are scored and ranked using a modified NIH-style trainee grant scoring system (Supplemental material). Reviewers consider academic qualifications, potential for a successful career in GBV/VAC-related research, quality and feasibility of the research proposal, ability of the candidate to bring a novel perspective and diversity to the researcher pool (e.g., women and other, under-represented gender-, sexual-, and ethnic-minority scientists), and relevance of the research proposal to the Vietnamese national research priorities around GBV and VAC. Rankings are provided to the CONVERGE Executive Committee, who make final decisions that consider reviewer rankings as well as diversity, balance of disciplines, and commitment of collaborating institutions to support the candidates upon completion of their training program.

#### Phase 1d: virtual onboarding and matching long-term post-doctoral trainees with research Mentor teams

Upon acceptance and before coming to Emory for the one-month intensive training, trainees complete a virtual onboarding and orientation with the DR and a collaborator in Vietnam. This virtual orientation covers the logistics of traveling to the US and connecting with Emory IT systems (email, outlook, online library system); an overview of training program goals and expectations; required deliverables of the one-month intensive, and timeline for completion. Selected trainees begin work on an NIH-style biosketch and continue development of their research proposals. The CONVERGE Executive Committee suggests suitable US research mentors based on the selected trainees’ application materials.

### Phase 2: CONVERGE mentor teams and mentorship training

#### Phase 2a: Pre-arrival creation of mentor teams

Following best practices for mentoring in the health sciences (80), the DR and MPI at HMU match each selected trainee with a mentoring team that includes: (1) a primary research mentor from their parent institution in Vietnam, (2) a primary, US-based research mentor with subject-area expertise; and (3) an early-career peer mentor with recent experience in a similar training setting. The mentor team is created immediately after selecting each long-term training cohort. This allows time for mentorship training before long-term post-doctoral trainees begin their training program. As needed, trainees also may consult other CONVERGE mentors who have relevant expertise.

US mentors take primary responsibility for training during the one-month intensive research training at Emory. In this capacity, US mentors shepherd the trainee through the intensive research training; guide the development of the trainee’s research protocol; identify and connect trainees with appropriate experts to enhance the quality of the research proposal; provide guidance and resources to improve scientific writing; and provide opportunities to develop the scholarly research network of the trainee (e.g., making introductions; identifying suitable conferences; publications). Female trainees are connected with mentors from the Women’s Research Career Development Sub-Committee (Figure 2) to receive additional support to succeed in environments that historically have been male dominated. In-country primary mentors take primary responsibility for the in-country mentored research project(s). These mentors provide ongoing scientific feedback and navigate institutional logistics relevant to the mentored research project; foster a collaborative research environment; supervise and critically evaluate research activities toward timely delivery of outputs; and offer guidance on career development opportunities. We routinely evaluate mentorship as part of our program evaluation to ensure a good fit between mentors and mentees and make course corrections, as needed.

#### Phase 2b: in-country mentorship training: rationale and eligibility

The success of each CONVERGE long-term post-doctoral trainee depends, in part, on strong in-country mentorship. Such mentorship involves guidance on trainees” research projects as well as support in acquiring academic positions, promotions, and recognition for professional achievements through nominations for awards and accolades (29, 81, 82). Although this kind of mentoring has been instituted throughout the US as a crucial aspect of research training, mentorship at academic institutions in LMICs can experience challenges, such as a limited number of qualified mentors, misunderstandings between mentors and mentees about roles and responsibilities, a lack of institutional support for mentoring, and little recognition for the value of mentorship for building research capacity (83–85). Furthermore, the effects of mentoring may not be apparent in academic outputs (81). To overcome these challenges, building mentoring capacity in LMICs is crucial to support collaboration among investigators, increase the quality and quantity of funded studies, enhance the research productivity of funded studies, and ultimately, strengthen research infrastructure (86). For these reasons, Drs. Katz and Glass at the National Institutes of Health (NIH), Fogarty International Center (FIC) have highlighted the general importance of comprehensive mentorship training in LMICs (87).

According to our needs assessment (Table 1), mentorship training remains a nascent part of research training in Vietnam. Therefore, CONVERGE emphasizes training in the knowledge and art of mentoring for mid-career research faculty at partner institutions in Vietnam, as well as for graduates of the long-term post-doctoral training program. Specifically, we offer mentorship training through a combined virtual and in-country short-course with 40 eligible short-term trainees. Eligible mentor trainees include approximately 25 mid-career faculty at partner institutions in Vietnam and approximately 15 long-term trainee graduates. Mentorship trainees should have (1) a strong research record, and ideally, experience in GBV/VAC research or an intersecting area in mental, physical, or behavioral health; (2) the desire to mentor trainees, and ideally, the research support and experience to mentor trainees; and (3) support from their institution to take part in 14 contact-hours of virtual and in-country mentorship-training learning modules. This approach to mentorship training creates a network of trained research mentors at diverse career stages, who can effectively mentor CONVERGE trainees and other aspiring researchers in Vietnam into the future, optimizing the sustained impact of CONVERGE.

#### Phase 2c: mentorship training curriculum

All short-term mentorship trainees complete a virtual 14-contact hour course before each 18-month, long-term postdoctoral training cycle. Mentorship training continues through virtual and in-country sessions during each 18-month postdoctoral training cycle. Virtual sessions include a mix of Vietnam-mentor only sessions and cross-cultural mentor training sessions with in-country mentors who are participating in other D43s supported by Emory University, focused on injury/violence prevention research, and underway in Southeast Asia. The mentorship training curriculum, summarized in Table 2, is adapted from the book, *Influential mentors: A guidebook for building mentoring skills and capacity,* developed with support from the NIH/FIC (D43TW010143-05S1; D43TW009127; D43TW007124) (88). This curriculum is based on adult learning theory and includes didactic training, panel discussions, group work, interactive exercises, and case studies. The curriculum has been implemented and favorably evaluated with Division Chairs at Calmette Hospital, Cambodia. In 2020–2021, due to the COVID-19 pandemic, Dr. Comeau adapted and implemented the curriculum virtually for five research programs, including several NIH/FIC-funded training programs (D43TW009127; D43TW010934; D43TW011404; D43TW007124).

**Table 2:** Mentorship training for CONVERGE mentors*.

Module and competency (81, 86)	Learning objectives	Instructional tools
Becoming a mentor *Competency:***Assess and provide skills and knowledge needed for success**	Describe the roles and responsibilities of mentors and cultural contexts Identify core values and characteristics of mentorship Identify mentorship goals	Review of mentoring models and frameworks Reflection questions and personal assessment Case study and discussion
Setting expectations between mentors and mentees *Competency:***Align expectations with reasonable goals and objectives**	Set expectations between mentors and mentees Use an Individual Development Plan (IDP) to identify and track mentee goals and progress Use a mentor-mentee agreement to guide mentoring relationships	Case study on mentor/mentee relationships Worksheets for individual development plans (IDPs) and mentor-mentee agreements Questions and communication for conversations on IDPs and mentor-mentee agreements
Communication for Influential Mentoring *Competency:***Maintain effective communication**	Complete DiSC communication style assessment Demonstrate skill in cross gender/culture communication Give actionable and effective feedback	Review results from DiSC communication assessment Identify communication styles and strategies Case study on difficult conversations; Roleplay
Mentoring Diverse Trainees *Competency:***Address diversity and culture**	Discuss impacts of gender, culture on mentoring practices Develop plans to practice self-reflection, overcome biases	Research on diversity and mentoring Reflection questions Case study on gender and mentoring Cultural humility model and worksheet
Sustaining Productive Mentees *Competencies:***Overcome resource limitations and Promote professional development and independence**	Describe the role of a mentee Identify the steps to independence Identify typical setbacks and solutions List resources to help overcoming research barriers Identify funding opportunities for mentees	Case study: mentee perspective Activity: Timeline to independence for postdocs and junior faculty Time management tools for mentees Identifying funding for GBV/VAC research careers
Building Mentoring Capacity *Competency:***Advocate for institutional change and support**	Develop steps for implementing/improving mentorship Identify resources to support mentoring Advocate for institutional support	Complete plan for implementing and improving mentorship Peer mentorship models Develop plan to sustain mentor training in GBV/ VAC research
Evaluating Mentorship *Competency:***valuate mentorship and mentoring programs**	Describe evaluation processes to ensure productive mentorship Develop evaluation plan for mentorship Review data collection tools	Evaluation case studies Worksheet: establishing metrics for evaluation Evaluation tools for mentorship, qualitative and quantitative assessment

Additional modules: Promoting professional integrity and ethical conduct; work-life balance; peer and group mentoring models; building mentoring teams; coaching and mentoring.

*Adapted from Comeau et al. (88), Hamer et al. (89), and Pfund et al. (90).

The core curriculum includes seven modules and competency areas on: (1) becoming a mentor; (2) setting expectations between mentors and mentees; (3) communication for influential mentoring; (4) mentoring diverse trainees; (5) sustaining productive mentees; (6) building mentoring capacity; and (7) evaluating mentorship (81). This curriculum includes skill-building to implement Individual Development Plans (IDPs) for trainees, mentor-mentee agreements, and checklists and timelines for program deliverables (Supplemental material). Some tools are adapted from prior NIH/FIC training programs and have been favorably evaluated by mentors and trainees (D43TW009127; D43TW007124; D43TW010143; D43TW010934; D43TW011404). The curriculum also involves completion of a working style assessment to identify communication and problem-solving styles as well as motivators and barriers to productive relationships and teamwork (91). This curriculum will be adapted for mid-career mentorship trainees to enhance topical knowledge of GBV and VAC research and to ensure suitability for Vietnam, based on feedback from the year-one evaluation.

#### Phase 2d: mentorship training delivery

The COVID-19 pandemic made travel to Vietnam for in-person training uncertain. Also, mid-career mentor trainees have fulltime professional obligations that can preclude multiday, in-person trainings. Thus, a combined virtual and in-person format for delivering the mentorship training curriculum over an 18-month cycle provides maximum flexibility to accommodate these contingencies. In general, we expect that mid-career mentor trainees will take part in four 90-min virtual sessions before the arrival of each cohort of long-term postdoctoral trainees. These sessions will cover three core topics: (1) foundations of mentoring (defining roles and responsibilities, recent research on positive outcomes of mentoring); (2) use of mentoring tools, such as individual development plans (IDPs) and mentor-mentee agreements; and (3) communication and working styles. To enhance learning on topic three, mentorship trainees complete the Myers Briggs personality test^1^ to reflect on the working styles of themselves and their mentees. After the one-month training intensive training is complete, the instructor holds additional virtual and in-country mentorship training sessions with mentors to ensure their use of mentoring tools adapted for CONVERGE and to cover the remaining topics in the adapted curriculum. To the extent possible and to allow for cross-cultural learning and professional networking, some of the virtual sessions are collaborative with mentors participating in other D43 training programs at Emory, in Southeast Asia, and focused on violence prevention. This flexible, extended format for delivery provides the additional advantages of time to make real-time course corrections and customizations for each cohort of mentor trainees, based on feedback from the evaluation. We will evaluate the mentorship training each year for this purpose.

### Phase 3: one-month training intensive of post-doctoral research fellows

#### Phase 3a: 1-month intensive research training: deliverables and curriculum

Each cohort of long-term trainees travel to Atlanta for a one-month intensive research training at Emory University to optimize the resources available to train independent investigators. Deliverables of this training include: (1) an NIH-style Specific-Aims page, (2) online CITI certification, and (3) a written research proposal that is presented and defended to an audience of peers and faculty for implementation during their 17-month in-country mentored research training period. Deliverables to be completed in Vietnam include an ethics/institutional review board protocol of their research project to be submitted at their home institution within 8–10 weeks upon completion of the one-month intensive training. To apply learning from course-work for higher-level synthesis and to meet the required deliverables, all course assignments enable trainees to make progress on their research proposal throughout the month-long training. Course instructors and Atlanta-based research mentors review all course assignments throughout the month-long intensive to ensure regular, constructive feedback that culminates in a draft research proposal. Key dimensions of this feedback focus on the relevance of their research project to current research on GBV and VAC and the feasibility of completing their project during their 17-month period of program support in-country. Following NIH guidelines, mentors ensure that the trainees’ research projects are rigorous, ethical, and considering the impact of age, sex, gender, and other differences in the design and analysis.

All course content is foundational to completing the course assignments, which lead to a rigorous research proposal. Table 3 provides a sample curriculum, and six core topics are covered: (1) concepts, measurement, and ethics in GBV and VAC research, (2) principles of mentee-mentor relationships, (3) responsible conduct of research, (4) research methods, (5) proposal development, and (6) managing and leading teams. The content of each topic, to some extent, is customized for each cohort, and core competencies associated with each topic are described, below.

**Table 3:** CONVERGE 1-month training intensive summary curriculum.

Week 1	Monday	Tuesday	Wednesday	Thursday	Friday	Deliverables
Morning 9–12	Intro, defining GBV, VAC theories, intersecting consequences	Measuring GBV	Measuring violence against children (VAC)	Ethics and best practices for GBV research	Ethics and best practices for VAC research	Draft specific aims Identify grant mechanisms
Lunch		Journal club				
Afternoon 1–4	Emory library orientation	Grant writing: Individual meetings	Mentee training	Working session: Specific aims	Simplifying scientific speech	
Week 2	Monday	Tuesday	Wednesday	Thursday	Friday	Deliverables
Morning 9–12	Mentee Training	Quantitative/Qualitative data collection (parallel sessions)	Qualitative/Quantitative data collection (parallel sessions)	Quantitative/Qualitative data collection (parallel sessions)	Working session: Specific Aims	CITI certificate Refine specific aims outline research approach
Lunch		Journal club				
Afternoon 1–4	Working session: Online CITI certification	Grant writing and workshopping	Working session: Research proposals	Funding and concept notes	Leadership Development	
Week 3	Monday	Tuesday	Wednesday	Thursday	Friday	Deliverables
Morning 9–12	Mentee Training	RCR Training (1 h) Qualitative/Quantitative data analysis (parallel sessions)	Working Session: Peer review draft research proposal	RCR Training (1 h) Qualitative/Quantitative data analysis (parallel sessions)	RCR Training (1 h) Qualitative/Quantitative data analysis (parallel sessions)	Full draft of research proposal (4 pages)
Lunch		Journal club				
Afternoon 1–4	Scientific Writing	RCR training	Grant writing	Intro to REDCap RCR training	Working session: Peer review draft research proposal	
Week 4	Monday	Tuesday	Wednesday	Thursday	Friday	Deliverables
Morning 9–12	Mentor Training	Grant Writing	Working session: Proposal revisions	Working session: Proposal revisions	Fellow proposal “Defense”	Final research proposal (4 pages) Research budget and justification (IRB for in country)
Lunch		Journal club				
Afternoon 1–4	Working session: Proposal, presentation	Working session: Proposal, presentation	Presentation Skills	RCR training	Visit to GSU	

RCR, responsible conduct of research.

##### Concepts, measurement, and ethics in GBV and VAC research

CONVERGE training faculty with specific expertise in GBV and VAC research cover foundational topics on (1) defining GBV and VAC, including specific forms, (2) etiological theories of GBV and VAC and intersecting mental, physical, and behavioral health outcomes, (3) measurement of GBV and VAC, and (4) ethical consideration and best-practices for conducting research on GBV and VAC. Content is tailored to focus on the forms of GBV/VAC and populations that are the foci of the trainees planned research projects. Topics also may include understanding violence as a public-health issue and translating data on GBV/VAC into action.

##### Principles of mentor-mentee relationships

The mentoring instructor and a collaborator in Vietnam facilitate at least one module on mentor-mentee relationships with CONVERGE trainees. The curriculum is based on Comeau et al.’s book, *Influential Mentors: Building Mentoring Skills and Capacity* (2019). The session provides foundational background and context on mentoring, a case study discussion on aligning expectations between mentors and mentees, and how to understand and incorporate feedback from mentors. Specific topics may include aligning mentor and mentee expectations, typical roles of a mentee, communicating with mentors, and trouble-shooting mentee missteps. The session also includes discussion on the cultural differences between mentors in Vietnam and the US, and strategies for the mentees to optimize mentoring relationships in the CONVERGE program. This session is designed to ensure that trainees interact confidently and coordinate well with their US and Vietnamese mentors.

##### Responsible conduct of research

NIH has established competencies in the Responsible Conduct of Research (RCR) (92). NIH mandates that this training be primarily face-to-face and tailored to the specific needs of the trainees. In this D43, which focuses on GBV and VAC, special attention is paid to the topics of Human Subjects in Research, and data security. RCR is addressed through multiple modalities. During the one-month intensive session at the Emory campus, at least five in-person sessions are provided, and if needed, the remainder are covered during annual in-country trainings. Topics include human-subjects research, ethics in data handling, authorship, maintaining participant privacy and data confidentiality, conflict of interest and commitment, mentor/mentee responsibilities and relationship, safe research environment, collaborative research, and research misconduct. Sessions are led by the Faculty Director for the Responsible Conduct of Research course, coordinated by the Office of Post-Doctoral Education within the Emory University School of Medicine. Mentorship is built into the overall training program, as described elsewhere in this paper.

##### Research methods

Course content is tailored to the specific needs of each trainee cohort. For trainee cohorts implementing projects mainly involving primary data collection, the focus of the one-month intensive sessions is on quantitative and qualitative field methods in GBV and VAC research. For trainee cohorts implementing projects mainly involving secondary data analysis, the focus is on the salient methods for quantitative and qualitative data analysis. Trainees also are exposed to useful software applications for data collection and/or analysis, such as REDCap or MaxQDA.

##### Research proposal development

These sessions guide trainees through the core steps of developing a research concept using publicly available data from their home country or collecting their own data during their in-country training. Topics focus on finding sources of federal funding, developing an NIH-style biosketch, crafting an NIH-style specific-aims page, and crafting the research strategies section of an NIH-style proposal. Didactic sessions are followed by “working sessions” in which fellows apply what they have learned to their own research proposals.

##### Scientific writing and science dissemination

A key component of strengthening research capacity among CONVERGE trainees is developing the communication skills needed to present and publish findings internationally. Therefore, foundational science dissemination sessions designed to support CONVERGE orientation program deliverables are included in the training curriculum. Sessions cover (1) scientific writing: paper-writing mechanics, peer review, predatory publishing, good scholarship practices, (2) presentation skills: slide design, presentation organization, public speaking, and (3) simplifying scientific speech: concise, effective communication across linguacultural contexts. These sessions are facilitated by training faculty who specialize in advancing global health research through applied linguistics.

##### Foundations of leadership

CONVERGE trainees are expected to manage a cross-cultural mentoring team, to develop and manage a project budget, and to develop and manage a project timeline. Yet post-doctoral trainees rarely receive formal training in such topics. In the absence of this training, people do the best they can; however, the process often takes longer with poorer results compared to those who receive foundational training. Post-doctoral fellows’ who receive such foundational training are well-positioned for success on these crucial leadership skills. CONVERGE training faculty with expertise in cross-cultural leadership development provide sessions on: managing teams, project management, influence without authority, and other leadership-related topics that are customized to each post-doctoral trainee cohort. We design experiential sessions that provide crucial leadership skills, yet also allow for some flexibility to serve the unique needs of each cohort.

#### Phase 3b: delivery of 1-month training intensive

The entire one-month training intensive is delivered at Emory University. In general, the delivery format involves in-person didactic (knowledge- and skills-based) sessions in the morning and interactive and working sessions in the afternoons (Table 3). All lectures are recorded, and all session materials and recordings are saved on Emory’s Canvas course platform, so trainees can access the materials anytime during the 18-month fellowship period. The final week of training applies the learning from the prior 3 weeks to finalize a draft research proposal and presentation, and to defend the research proposal with peers and faculty at the end of the intensive. The delivery of the one-month training adheres to the planned protocol, described above, with flexibility for revisions to meet the specific interests and skill domains of each cohort of long-term CONVERGE trainees. For example, we may add additional sessions on the responsible conduct of research; the use of REDCap as a secure system for the collection, transfer, storage, and analysis of study data; and finding grant funding from foundations. We also may revise the final set of deliverables to accommodate the initial skill levels of each cohort of trainees.

### Phase 4: mentored research project in Vietnam and concurrent training

#### Phase 4a: IRB protocol development

After completing their research protocol and before beginning their research project, these research protocols must receive the Institutional Review Board (IRB)'s approval in Vietnam. The IRBs at partner institutions in Vietnam are available for the fellows to apply. The fellows submit all institutional forms, as needed, which may include research protocols, questionnaires, and other documents, like informed consent forms that provide participants with information about potential benefits and risks. The IRB committee must receive application materials in Vietnamese for approval. When the draft IRB application is complete, the fellow’s primary mentor in Vietnam and a CONVERGE collaborator at HMU review all IRB applications before submission. If there are any modifications to the research protocol while it is being implemented, they must be submitted to the IRB Committee for approval before moving further. The trainee can start data collection after receiving the IRB certificate.

#### Phase 4b: on-going support of fellows’ research progress

Several strategies are in place to support the completion of long-term trainees’ mentored research projects. First, trainee develop a project timeline for self-checking of the timely completion of project milestones (Supplemental material). Second, trainees organize weekly meetings with in-country research mentors to provide real-time guidance on project implementation. Third, trainees organize monthly meetings with their mentor teams so conceptual, methodological, or operational challenges that may emerge can be addressed with the appropriate expertise. Fourth, the DR and an in-country collaborator host monthly check-ins to identify and address any challenges with mentoring teams or projects as they arise. Fifth, the Emory project coordinator administers a brief REDCap survey to trainees to ensure mentor meetings are occurring and meeting minutes are documented for evaluation. Sixth, a regular journal club and annual in-country trainings are provided to advance the research and dissemination skills of trainees. Finally, periodic virtual sessions are hosted by the CONVERGE mentorship instructor or other program faculty to support the professional development and networking of trainees supported across D43s at Emory, in Southeast Asia, and focused on injury/violence prevention.

#### Phase 4c: deliverables of the CONVERGE fellowship

Trainees are expected to complete two primary deliverables by the end of their 18-month fellowship. These deliverables include (1) the submission of an abstract and the presentation of research findings at an international scientific meeting, and (2) the drafting and submission of one research article to an international, peer-reviewed journal. The format of the research article depends on the nature of each trainee’s research project (e.g., qualitative versus quantitative) and is expected to follow the guidelines of the journal to which they submit. Finally, 6 months after the completion of their 18-month fellowship, trainees are expected to submit a second proposal, to a competitive funding pool within CONVERGE, to a private foundation, or to NIH. This follow-up proposal should build on the findings from their CONVERGE fellowship and further advance their research training toward becoming an independent investigator and future mentor of students at their respective institutions.

### Phase 5: concurrent monthly journal club during research project implementation

A CONVERGE journal club begins during the one-month intensive training (Table 3) and continues virtually during the trainees’ in-country mentored research projects. The journal club is designed to build a peer-network of GBV/VAC researchers across partner institutions in Vietnam. It also provides a space to discuss articles on GBV or VAC that are timely, high-impact, of interest to the trainees, or cover advanced research methods that trainees might consider in their own GBV/VAC research projects. Building on the format of existing journal clubs within Emory doctoral programs and other D43 training programs, sessions are small and informal, but structured to encourage discussion and questions, enable critical evaluation of the research design and manuscript, and ultimately, inform the work of the trainees. Sessions are one-hour and held monthly using an in-person or virtual format. One trainee is responsible for leading each journal club session. Leadership includes selection and circulation of a peer-reviewed article among the trainee group 2 weeks in advance of the journal club, presenting a summary and critical evaluation of the article during the journal club, and responding to questions that arise from other trainees. Leadership also includes preparing a short PowerPoint presentation the includes: (1) a brief description of why they selected the article, (2) the research question and study aims, (3) the study design (including unique ethical considerations given the GBV/VAC research context), (4) a summary of the major findings and discussion points, (5) a brief statement of how the study impacts their own future work, and (6) 2–3 questions for the group to discuss. The other trainees read the article in advance and come prepared to discuss the article in detail. The lead trainee for the journal club moderates the discussion, and a faculty member is present for each journal club to answer questions and to ensure critical points from the article are discussed.

### Phase 6: strategies to ensure long-term postdoctoral trainee retention

CONVERGE provides several sources of support to promote trainee retention. First, we create multiple-mentor teams, with appropriate expertise and a structure in which long-term trainees meet weekly with trained in-country mentors and monthly with US mentors and mentor teams. Second, trainees participate in a customized one-month intensive course at Emory University in the United States, which allows trainees to receive needs-based training and to develop in-person, professional relationships with their US mentors. Third, trainees receive a monthly stipend to allow for protected time for mentored research and research training in the CONVERGE program. Fourth, trainees receive research funding to support their proposed in-country research project. Fifth, trainees receive support for computing and an Emory email account, which provide them with access to Emory electronic resources including e-journals, e-textbooks, and proprietary funding resource databases. Sixth, trainees receive multiple, ongoing opportunities for training during their 17-month in-country research project, such as an on-going virtual monthly journal club, annual in-country training sessions, and virtual networking opportunities with other D43 trainees in other countries. Seventh, trainees have opportunities to share confidential feedback to the CONVERGE evaluation team for real-time course corrections, and feedback during regular check-in meetings with the Director of Research. This feedback enables trainees to communicate challenges they are having in the CONVERGE program that the leadership team can discuss and address proactively. This combination of human capital investment, professional network development, and financial investment builds on the PI’s experience with empowerment-based training of under-represented early-career scientists (29), and should result in a high retention rate of long-term postdoctoral trainees in the CONVERGE program, as well as continued GBV/VAC research after program completion.

### Phase 7: advanced in-country trainings and developing a science-to-practice network

In Years 4 and 5 of the project period, CONVERGE will host in-country trainings that will advance the methodological, leadership, and science-dissemination skills of all three cohorts of long-term trainees. One short-course will focus on the concepts, theories, and methods of implementation science. A second short-course will focus on advanced topics in leadership training, and a third short-course will focus on skills and strategies for various types of science dissemination (89, 90).

### Implementation science

Training in implementation science will take the form of a short course on Dissemination and Implementation (D/I) science. Course content will start with a review of theoretical perspectives on D/I science and will cover several current theoretical models, including Diffusion of Innovation (93), RE-AIM (Reach, Effectiveness, Adoption, Implementation, Maintenance) (94), EPIS (Exploration, Preparation, Implementation, Sustainment) (95), and CFIR (Consolidated Framework for Implementation Research) (96). The course will cover methodology of D/I research including etiological methods, methods from D/I trials, and combined effectiveness/implementation trials, and the use of mixed methods in D/I research (97). Another module will focus on measurement of key constructs and will review methods for identifying common outcomes in D/I research (98). Additional content will be focused special topics including systemic and organizational influence on D/I (99), the use of technology in D/I research (100), processes for adapting evidence-based programs (101), sustainment of implementation (102), and “real-world” implementation conducted in a non-research setting.

### Advanced topics in leadership

This short-course will build on the foundational topics covered in the one-month intensive—on managing projects, leading teams, and influence without authority. Advanced topics in leadership development will include, for example, making ethical decisions through emotional intelligence, effective negotiation, and conflict management. The goal in the advanced course is to help our trainees build skills to manage issues that commonly arise in the process of team projects. We will design the content to help participants recognize early when team issues arise, diagnose the nature of the problems that have surfaced, and tactfully communicate with their teams to overcome the challenges. Conflict is a key example, as many research teams struggle to resolve differences of opinion, divide responsibilities thoughtfully across team members, and other process issues related to working with one another. These challenges can lead to team dysfunction and damaged relationships among its members, thus a focus on emotional intelligence to attend to the relationships among team members. The short course also will cover the qualities and skills needed to become a transformational leader, leadership qualities needed to spearhead intra-organizational change, and strategies to manage cross-cultural organizational collaborations. After participating in these sessions, post-doctoral trainees will be more savvy and holistic proponents of change, including team members in the process and ensuring that all voices are heard in the plans and implementation of ideas.

### Advanced topics in science dissemination

This short course will build on the foundational topics covered in the one-month intensive by focusing on strategies for communicating scientific research findings effectively across diverse contexts and audiences. In this task-supported course, trainees will develop in-progress research presentation and writing projects through hands-on, peer-review activities. Topics include simplifying scientific speech, presenting at international conferences, communicating across cultural contexts, and composing professional correspondences. Trainees will develop strategies for crafting concise and compelling abstracts, posters, and PowerPoint presentations for international scientific conferences. The course will explore intercultural conventions for professional communications such as emails, cover letters, revisions, and revise-and-resubmit responses. It also may focus on skills for developing effective press releases about the findings of research projects and interacting effectively with local, national, and international media about the findings of research projects. Finally, this short course also may cover strategies for communicating with local, regional, and national officials to convey the findings of research projects in concise, lay terms.

### Scientist-practitioner network

CONVERGE also will support the development of an in-country science-to-practice network to optimize the public-health impact of the program. To do so, we will offer these in-country short-courses to 60 practitioners and to our growing pool of research mentors. Eligible short-term practitioner trainees will include professionals working in universities, schools, community-based organizations, healthcare systems, and political organizations that are salient for the GBV and VAC evidence being generated by CONVERGE trainees. Examples include those working in mother and child protection in the Department of Health and Human Services; Child Protection Department, Ministry of Labor, War Invalids, and Social Affairs; Maternal and Child Health Protection Department, Ministry of Health; Vietnam woman’s group; and Child Rights Working Group, a consortium of international and domestic organizations who cooperate and share information to promote children’s rights in Vietnam. Eligible practitioner trainees should (1) have a history of working on policies or programs related to GBV/VAC or a cognate field, such as mental health or sexual health; (2) be sensitive to the vulnerability of certain groups in Vietnam to the risks of experiencing GBV and VAC, (3) have an interest in the implementation of evidence-based GBV and VAC programs, and (4) have an interest in science-practitioner collaboration. We also provide mentorship training to practitioners. In project year 2, CONVERGE leadership will conduct needs assessment meetings with a purposive sample of practitioners to determine the most suitable topics and format of the training. Possible topics include: how to mentor new professionals who are entering a career in GBV and VAC, mentoring staff on how to manage competing priorities; mentoring professionals through essential and new skills required in the field; and, mentoring professionals on self-care as they work on topics and research that require emotional work.

### CONVERGE training program evaluation

To accomplish our program aims, CONVERGE will implement a rigorous, multi-level evaluation to assess the implementation, reach, fidelity, and effectiveness of the CONVERGE training program. Guided by an established evaluation framework and detailed evaluation plan, our evaluation will accomplish three specific goals: (1) to provide data for decisions that enhance modification and improvements of CONVERGE program activities; (2) to determine the individual and aggregate success of program activities and aims; and (3) to identify strategies for long-term sustainment and expansion of in-country research training to address GBV and VAC in Vietnam. The program evaluation is carried out under the direction of the Director for Evaluation (Figure 2).

### Evaluation framework

**Figure 3:**
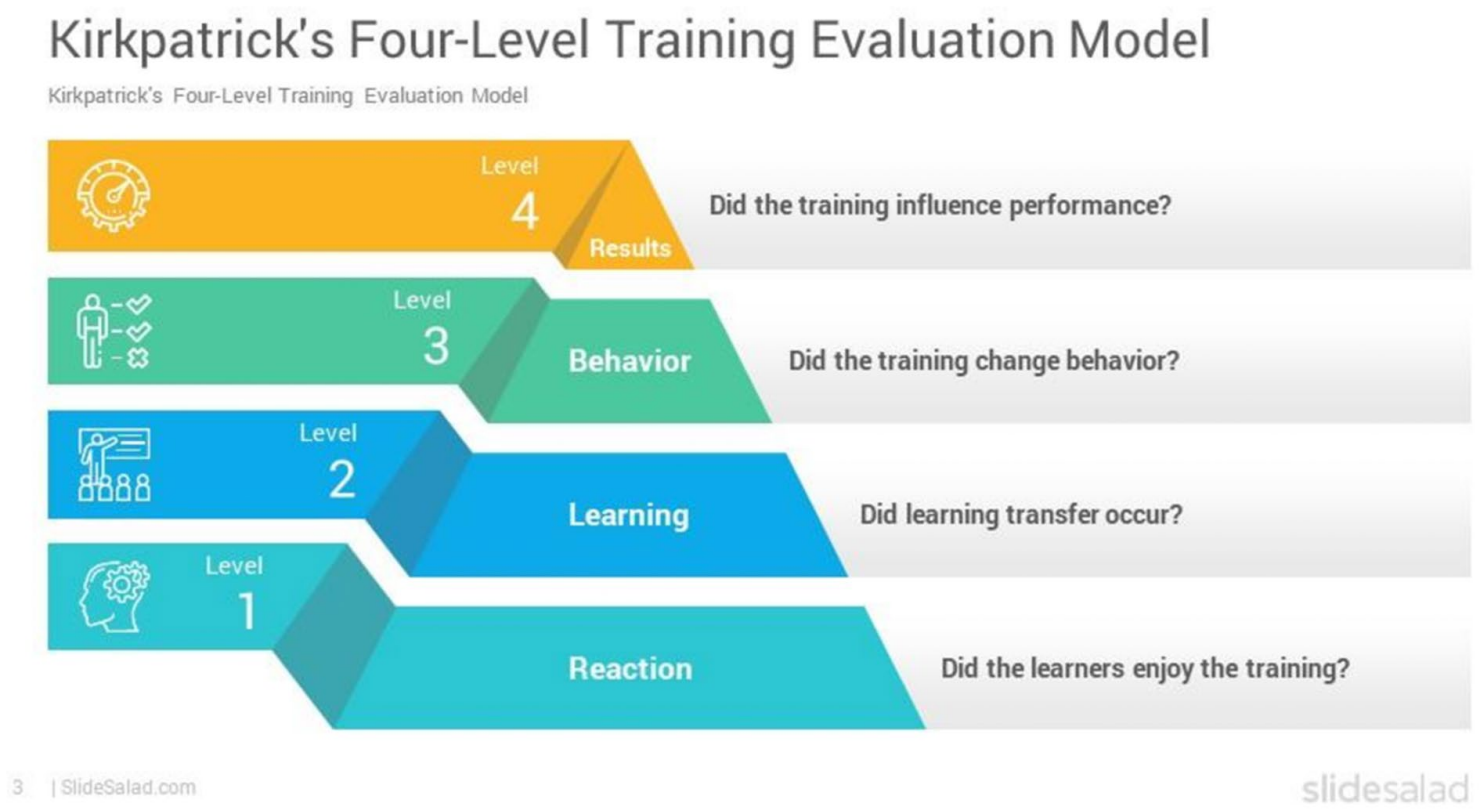
Kirkpatrick four-level training evaluation. Licensed for inclusion from slidesalad.cpm https://www.slidesalad.com/our-licenses/.

To achieve these goals, the evalution of CONVERGE is guided by the Kirkpatrick Model (103), an established framework for evaluating training programs in health care and international settings (104, 105). The Kirkpatrick model employs four levels of evaluation of training programs (Figure 3). The first level, *Reaction*, refers to the level of reaction by learners to the training. A measure of reaction is the degree to which training participants find the training favorable, engaging, and relevant to their jobs (106, 107). The second level, *Learning,* refers to the type and scope of changes in the participants’ learning caused by participation in the program. We can measure this level by addressing the degree to which the participants acquire the intended knowledge, skills, attitudes, confidence, and commitment based on their participation in the training. The third level, *Behavior,* indicates whether or not the program has created a desired change in the participants’ behavior. A measure of behavior is the degree to which participants apply what they learned during training when they are back on the job (108, 109). The fourth level of the Kirkpatrick Model is Results. This level reflects the degree to which targeted outcomes occur as a result of the training. These outcomes may be directly related to the individual’s work or the work of the organization (110).

### Data collection

We will use a mix of quantitative methods (surveys, course evaluations) and qualitative methods (in-depth interviews, focus group discussions) to assess trainee and mentor experiences with the program, achievement of program competencies, and progress with GBV and VAC research. We will adopt a bi-directional approach to evaluation, in which we obtain feedback *from trainees and mentors* about the program and assess *trainee and mentor progress*. Data collection instruments are developed and informed by each of the Four Levels of the Kirkpatrick Model.

Quantitative surveys and course evaluations are developed and deployed using the Research Electronic Data Capture (REDCap) system, a secure web platform for building and managing online databases and surveys. Its streamlined process for creating and designing projects offers an array of customizable tools for a specific data-collection strategy. Quantitative data is analyzed using descriptive, and as the number of CONVERGE trainees grows, multivariate statistical techniques. Qualitative methods include in-depth interviews and focus group discussions conducted with trainees and their mentors. Data collection occurs in person during annual in-country training and planning meetings as well as online via Zoom and email. Using a team-based approach, all qualitative data will be coded, and standard content analysis will be applied to develop themes. Triangulation of the quantitative and qualitative data will occur during a stage of final interpretation. Evaluation data will be collected continuously over the program period and used iteratively to inform decisions to improve program content, design, delivery, and implementation. See Supplemental material for sample evaluation forms.

### CONVERGE evaluation logic model

**Figure 4:**
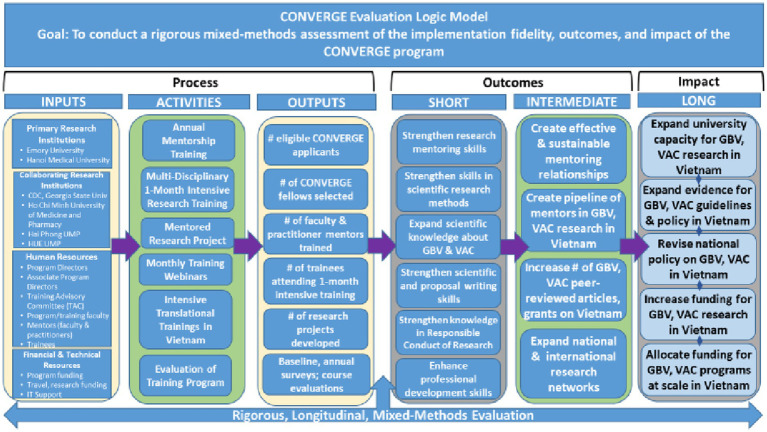
CONVERGE evaluation logic model.

We developed a logic model that operationalizes the Kirkpatrick model and provides a guiding framework to evaluate CONVERGE. This logic model provides a systematic and visual guide to the relationships that exist among resources allocated to the CONVERGE program, planned programmatic activities, and anticipated changes as a result of the program. We developed this logic model to refine: (1) the focus of the evaluation; (2) the evaluation questions, or what is to be measured; (3) the indicators and information that best answer the evaluation questions; (4) timing for data collection; and (5) types of data to be collected using appropriate sources, methods, samples, and instruments (Figure 4). The evaluation logic model is organized by process, outcome, and impact measures. Process measures are those that identify the inputs or resources, activities, and outputs of a program that are needed to ensure successful implementation of a program. Outcome measures include short-term and intermediate-term outcomes that are expected once successful implementation occurs. Finally, impact measures identify effectiveness of achieving a program’s long-term goals.

### CONVERGE evaluation plan

We developed an evaluation plan to help document program effectiveness and to inform decisions about future program development and/or needed improvements (Table 4). This evaluation plan identifies and describes each component of the training program to be evaluated and maps each component to the relevant evaluation measures, and data-collection activities. Details of each evaluation component are described below.

**Table 4:** Evaluation plan for the CONVERGE research training program.

Domain	Measure	Purpose	Data collection activity
Program enrollment and participation	*Process* Implementation Reach	Assess the enrollment and participation rate and characteristics of mentors and trainees Assess the enrollment and participation rate and characteristics of institutions sponsoring trainees	Document review of CONVERGE training and course content Baseline and annual quantitative surveys of mentors and trainees
Mentor and mentee trainings *Mentee training* *Faculty mentor training* *Practitioner mentor training*	*Process* Reach Implementation Fidelity *Outcome* Mentor Trainee impact	Measure success of course learning objectives and quality of instruction Evaluate course quality, strengths, weaknesses Assess mentor, mentee satisfaction with training Draft, review, revise, finalize mentoring deliverables	End of course evaluations Pre/post surveys of mentors and mentees Individual Development Plans (IDPs) Mentor-mentee agreements Qualitative in-depth interviews with mentors and trainees
Month-long intensive research training	*Process* Reach Implementation Fidelity *Outcome* Mentor Trainee impact	Measure success of course learning objectives and quality of instruction, specific to the core competencies (see Phase 3a and Table 3) Evaluate course quality, strengths, weaknesses Measure professional activities completed, courses completed, and research outputs (e.g., research proposals, IRB submissions)	Pre/post quantitative surveys of trainees Mid-course and end-of-course evaluations Course attendance/changes in attendance IDPs Qualitative in-depth interviews with trainees and mentors
Professional Development Training Activities *(network meetings, journal club)*	*Process* Reach Implementation, Fidelity *Outcome* Trainee impact	Assess participation in professional training activities Assess satisfaction with professional training activities Assess quality, frequency of mentor interaction	Post-activity quantitative surveys with trainees Qualitative in-depth interviews with trainees
17-month in-country mentored research projects	*Process* Reach Implementation Fidelity *Outcome* Mentor Trainee impact	Track mentoring and training activities Research outputs (e.g., grant applications submitted and awarded, manuscripts submitted, articles published, conference abstracts submitted and published) Measure professional activities completed (research project, grant submission, manuscript, conference abstract, conference presentation, follow-on research proposal)	Research project activities, including weekly in-country mentor meetings, monthly mentoring meetings, seminars, field trips and other training activities Individual development plans (IDPs), mentor-mentee agreements, checklist of deliverables Conference abstract and presentation
Implementation science short course	*Process* Reach Implementation Fidelity *Outcome* Trainee impact	Assess number/scope of attendees, overall and by profession, including early-career fellows, research mentors, and practitioners Measure knowledge, efficacy, and practice of implementation science research practices, overall and by profession	Pre/post quantitative surveys of trainees Course evaluations
Leadership and Science Dissemination training	*Process* Implementation Reach Fidelity *Outcome* Mentor Trainee Institutional impact	Assess number/scope of attendees, overall and by profession, including early-career fellows, research mentors, and practitioners Measure knowledge, efficacy, and practice of leadership and research dissemination practices, overall and by profession Measure expansion of long-term professional network and collaboration (e.g., number of co-authors on presentations, publications, grants)	Pre/post quantitative surveys of trainees, mentors, and program partners Course evaluations IDPs Research dissemination presentations
GBV and VAC research capacity in program/translational research and training	*Outcome* Mentors Trainee Institutional impact	Measure the translation of mentored research projects into practice and public policy Measure dissemination of research findings (number of local dissemination events, international scientific conferences, policy meetings with CONVERGE fellows presenting)	Document review to assess new country and institutional policies/laws that are passed/ implemented as a result of our trainees’ GBV research and to capture dissemination of findings through communication channels (social media, other media, written communication) IPDs Focus groups with trainees, mentors, program partners/institutions
Long-term impact	*Outcome* Mentor Trainee Institutional impact	Proportion of trainees engaged in research Increased GBV/VAC research capacity in Vietnam, new collaborations, multidisciplinary research Number of publications in PubMed journals Number of GBV/VAC-related grants submitted and awarded to current and former trainees and collaborating institutions Increase # of high-quality mentors in Vietnam; (former) trainees as mentors	Annual surveys and exit interviews of trainees and mentors Trainee IDP/CVs Qualitative in-depth interviews with trainees, mentors, program partners/institutions

### Program enrollment and participation

To assess program implementation and reach, we distribute quantitative surveys to track mentor and trainee enrollment and progress and engagement of US/international, Vietnam program partners and institutions. Ongoing monitoring of the program is conducted through a systematic review and updating of CONVERGE program and training materials.

### Mentor and mentee training evaluations

We collect multiple sources of data to evaluate the impact of the mentor and mentee trainings on mentoring practices and perceived competence in mentorship. We evaluate the faculty mentor trainings as well as the practitioner mentor trainings. For both mentor trainings, we develop and distribute pre- and post-training surveys of mentors to gauge changes in knowledge, attitudes, and beliefs regarding the value and understanding of mentorship. We conduct qualitative in-depth interviews with select mentors to explore further their experiences with the mentor training and perceived competence in mentorship. Our evaluation of the mentee training includes pre- and post-training surveys of mentees to gauge their knowledge, attitudes, and beliefs regarding the value and understanding of mentorship and the mentee-mentor relationship. We also conduct an end-of-course evaluation of the training to gauge satisfaction with the training and recommendations for mentee training enhancement. Finally, we conduct qualitative in-depth interviews with mentees to explore further their experiences with the mentee training and their mentoring relationships.

### One-month intensive research training

Our evaluation of the one-month intensive research training assesses success with course learning objectives, perceived quality of instruction, satisfaction with the training, and progress with professional development and research activities, such as the research proposal and IRB submission. We distribute pre- and post-training surveys to gauge changes in trainee knowledge, attitudes, and beliefs about GBV/VAC research. We also conduct mid-course and end-of-course evaluations of the training to gauge satisfaction with the training and recommendations to enhance mentee training. Finally, we conduct a select number of qualitative in-depth interviews with the trainees to explore their experiences with the month-long intensive, and with the mentors to assess trainee’s professional progress.

### Professional development training activities

During the one-month intensive research training, trainees also attend several weekly professional development activities, including mentor meetings, peer network meetings, journal club, and project management. We evaluate each activity independently and at the end of the month-long intensive research training. Trainees complete a post-training activity survey to assess participation in, satisfaction with, and knowledge gained. We also conduct qualitative interviews with select trainees to explore experiences with training activities and recommendations for program improvement.

### Seventeen-month in-country mentored research project

To monitor trainees’ progress with their in-country mentored research project, we track their attendance and participation in (1) weekly in-country mentor meetings; (2) monthly U.S/in-country/alumni mentor team meetings; (3) virtual training seminars; (4) review meetings to provide advice and support with executive committee members from HMU; and (5) innovation field visits to GBV/VAC research centers. We also analyze trainees’ IDPs and monthly mentor meeting notes. Trainees also are required to submit their research conference abstract and presentation to the evaluator.

### Implementation science short course

We evaluate this short course implemented in Year 4. We conduct pre- and post-training surveys to assess changes in knowledge and skill, as well as satisfaction with the training. We conduct an end-of-course evaluation to gauge satisfaction with the course and recommendations for program improvement. We also conduct qualitative in-depth interviews with trainees to gain further insight about the training as well as recommendations for program improvement.

### Leadership and science dissemination training

We evaluate the leadership training and science dissemination training in Year 5. We conduct pre- and post-training surveys to assess changes in knowledge, skill, and satisfaction with the trainings. These surveys incorporate measures of leadership with which the team is familiar (111). We also conduct an end-of-training evaluation to gauge satisfaction and recommendations for program improvement. Furthermore, we conduct qualitative in-depth interviews with trainees, mentors, and program/institutional partners to gain additional insight about the training as well as recommendations for program improvement. Copies of the trainee’s research dissemination presentations also will be collected, as well as their IDPs to assess the impact/translation of the training on trainees’ research and career development.

### GBV and VAC research capacity in program/translational research and training

A long-term goal of CONVERGE is to build capacity of trainees to conduct research related to GBV and VAC, in Vietnam and with their practice partners and institutions. We monitor the reach and translation of GBV and VAC research conducted by CONVERGE trainees through a review of peer-reviewed and gray (non-academic) literature. We assess the impact of this research on changes in national and local policy through additional document reviews and an inventory of engagements with in-country policy-makers. Focus group discussions are to be conducted and organized by group (mentor, trainee, program partners/institutions) to elicit further information about the research and translation of GBV/VAC research developed in the program.

### Long-term impact

To measure attainment of program goals and long-term program impact, we assess outcomes of individual trainees, mentors, and institutions participating in the CONVERGE program. Individual trainee outcomes include publications, grants, new roles in CONVERGE and at their home institutions. Mentor outcomes include increased mentor competence and new roles in CONVERGE and at their home institutions. Institutional outcomes for in-country institutions include increases in institutional GBV/VAC grants secured, number of articles on GBV/VAC produced, and spillover efforts to faculty outside the CONVERGE program. Several data sources are used to inform the evaluation of outcomes and long-term impact of the program, including quantitative surveys, annual and exit interviews (mentors and trainees), IDPs/CVs to track trainee publications and grants, and qualitative interviews with trainees, mentors, and institutional leaders.

## Discussion

### Summary of CONVERGE training program

Gender-based violence (GBV) and violence against children (VAC) are prevalent and interconnected global health challenges; however, data and research capacities to study these forms of violence and to generate evidence-based policies and programs remain limited in LMICs, including Vietnam. To address these shortages in Vietnam and to establish a model for other LMICs, we are establishing CONVERGE—the Consortium for Violence Prevention Research, Implementation, and Leadership Training for Excellence. CONVERGE connects experts from Emory University, HMU, partner institutions in Atlanta and Vietnam, and partners from around the world, with the goal of providing mentorship opportunities for early-career scientists in GBV and VAC research and prevention. Based on a needs assessment with partners in Vietnam, the CONVERGE training program has four components: (1) 14 h of virtual/in-person annual mentorship training to prepare research mentors and to create a pipeline of future mentors in Vietnam; (2) a one-month intensive research training for long-term postdoctoral fellows at Emory University; (3) a structured 17-month, in-country mentored research project for long-term trainees that results in a peer-reviewed manuscript and a grant submission; and (4) annual in-country trainings, including advanced training on implementation science, leadership, and science dissemination. CONVERGE will support 15 long-term, postdoctoral trainees with multi-disciplinary research training in GBV and VAC, 40 short-term mid-career academic trainees, and 60 short-term practitioner/stakeholder trainees over 5 years to build productive GBV and VAC academic, scientific, and practitioner networks. CONVERGE also will connect long-term trainees and mentors to peers who are participating in other D43s focused on violence/injury prevention, D43s at Emory, and D43s in Southeast Asia. To assess the reach, implementation, fidelity, and effectiveness of these four training components, we will implement a rigorous, mixed-methods, multi-level evaluation using process and outcome measures. Evaluation findings will be used to refine program components for future trainee and mentor cohorts and to assess long-term program impact.

### Implications for capacity-strengthening, evidence generation, and public-health impact

CONVERGE aligns with national research priorities of Vietnam (28) and leverages over 12 years of collaboration between consortium partners in Atlanta and Vietnam, demonstrating the high feasibility of achieving our program aims. Our mentorship training, which emphasizes trainee empowerment (29), transparent mentor-mentee collaboration, and team-based mentoring with trained mentors, will create a pipeline of early- and mid-career researchers who are skilled mentors. Our long-term post-doctoral research training is grounded in successful integrated, interdisciplinary training programs with Emory and other partners and is tailored to meet identified needs in Vietnam. Our advanced leadership, science dissemination, and implementation science short courses address documented needs in Vietnam (30), build on and amplify Emory’s successful leadership training programs (D43TW009135), and will accelerate the translation of evidence into contextually relevant policies, programs, and best practices. Our training plan addresses the priorities of several divisions within the National Institutes of Health (NIH) related to women’s health, child health and human development, mental health, minority health and health disparities, alcohol abuse and alcoholism, drug abuse, and general medicine. Our intentional integration of GBV and VAC research with intersecting health conditions and health disparities creates a platform for future grant submissions by CONVERGE trainee graduates.

Several events illustrate the favorable political climate for CONVERGE trainees to have favorable impacts in Vietnam. First, on January 13, 2021, the Prime Minister of Vietnam authorized the Vietnamese national program on avoiding and combating domestic abuse until 2025. Second, in a joint statement on January 5, 2022, the Child Rights Working Group committed to implement child protection measures in Vietnam and to support the Vietnamese government in its efforts. Third, on November 14, 2022, the National Assembly of Vietnam approved a revised Law on Domestic Violence Prevention and Control. This climate will enable CONVERGE trainees to collaborate with practitioners, organizational officials, and local advocates to make evidence-based recommendations to improve prevention and response services for those most vulnerable to GBV/VAC in Vietnam.

Our team anticipates potential pitfalls and unintended effects of CONVERGE for GBV/VAC research and practice in Vietnam. First, fellows’ research projects are selected with care to ensure local acceptability, given the potential sensitivity of topics related to GBV/VAC. Second, efforts are invested to expand the pool of in-country mentors to avoid local mentor burnout and to expand capacity for mentoring across all local partner universities. Third, long-term trainees and local mentors often have competing demands on their time, which may contribute to delays in the completion of trainees’ research projects. To mitigate this risk, CONVERGE has developed supports and “cross-checks” to ensure that project milestones are met or appropriately revised and that trainees cultivate effective problem-solving skills when they encounter time-related demands that may delay their progress. Fourth, fellows may lack the local professional networks to ensure the timely translation of their research into practice and policy with local influencers. To facilitate the favorable local impact of their research, the CONVERGE Executive Committee has integrated opportunities into the 18-month fellowships for trainees to present their work sequentially to the CONVERGE Executive Committee, subsequent cohorts of trainees, and influential local practitioners at annual in-country meetings. In these ways, and others, CONVERGE includes safeguards against unintended consequences of the program to ensure its long-term sustainment.

For these reasons, CONVERGE members are energized to meet the demand for locally relevant violence prevention research, implementation, and leadership training in Vietnam. Over the program period, we expect to train a critical mass of 115 research mentors, post-doctoral fellows, and practitioners in Vietnam in GBV and VAC research, implementation/dissemination research and practice, and leadership. Based on learning from prior training programs as well as novel training elements, we will promote high trainee retention and strengthen institutional capacity in Vietnam, with a strategic focus on CONVERGE partner institutions for maximum institutional impact. In doing so, we will prepare a consortium of universities and experts in Vietnam to conduct locally relevant GBV and VAC research and to translate evidence into recommendations that shape national research and program priorities into the 21^st^ century.

### Program adaptations to mitigate the impact of the COVID-19 pandemic

CONVERGE is poised to adapt all programming from in-person to virtual formats, to be responsive to the uncertainties of in-person meetings resulting from an on-going COVID-19 pandemic. At the same time, our team recognizes that training in the responsible conduct of research requires a high degree of in-person training, based on guidance from the NIH (92). Therefore, in-person training in the responsible conduct of research will be prioritized in our one-month training intensive and our in-country training meetings to meet NIH requirements. Otherwise, in-person program components may be adapted to be sequential, hybrid (in-person; virtual) formats or entirely virtual, as needed. Transitioning to a virtual format also may require extending the duration of training over a longer period to ensure adequate coverage of material. These contingencies will be considered to ensure that each group of trainees receives high-quality, comprehensive training in relevant areas of expertise.

## Conclusion

CONVERGE—Consortium for Violence-Prevention Research, Implementation, and Leadership Training for Excellence—is an innovative D43 training program and multi-institutional partnership led by Emory University and HMU that supports the development of a national network of scientific mentors, the career development of three cohorts of post-doctoral fellows to become scientific leaders, and institutional capacity strengthening in implementation research, science dissemination, leadership, and best practice in the prevention of gender-based violence and violence against children. The program benefits from a comprehensive mixed-methods evaluation plan that provides real-time feedback for continual process improvement. CONVERGE aspires to achieve the long-term impacts of expanding the evidence base in these thematic areas in ways that increase the allocation of funding to contextually relevant, efficacious violence-prevention programming that contributes to sustained, positive public-health impacts in Vietnam.

## Ethics statement

Ethical review and approval are not required for this study in accordance with the local legislation and institutional requirements. The participants will provide their written informed consent to participate in this study prior to their participation.

## Author contributions

KY: conceptualization, methodology, resources, investigation, writing—original draft, writing—review and editing, visualization, supervision, project administration, funding acquisition. SB: writing—original draft, writing—review and editing, visualization, methodology. DC and JS: writing—original draft, writing—review and editing, methodology, project administration. HV: funding acquisition, project administration, supervision, writing—original draft, writing—review and editing. AK: writing—original draft, methodology (of journal club), project administration. HN, IB, DP, MS, and AS: writing—original draft, writing—review and editing. All authors contributed to the article and approved the submitted version.

## Funding

This work was supported by the National Institutes of Health, Fogarty International Center International, training grant number D43TW012188 (PI KY; MPI Giang). Provided monetary support for the CONVERGE training program’s implementation, completion, and evaluation.

## Conflict of interest

The authors declare that the research was conducted in the absence of any commercial or financial relationships that could be construed as a potential conflict of interest.

## Publisher’s note

All claims expressed in this article are solely those of the authors and do not necessarily represent those of their affiliated organizations, or those of the publisher, the editors and the reviewers. Any product that may be evaluated in this article, or claim that may be made by its manufacturer, is not guaranteed or endorsed by the publisher.

## Footnotes

^1^ https://www.myersbriggs.org/my-mbti-personality-type/mbti-basics/

## Glossary

CDC: Centers for Disease Control and Prevention
CRIV: Center for Research on Interpersonal Violence
CEFM: Child, early, or forced marriage
CFIR: Consolidated Framework for Implementation Research
CONVERGE: Consortium for Violence-Prevention Research, Implementation, and Leadership Training for Excellence
D/I: Dissemination and Implementation
SCORE: Emory Specialized Center for Research Excellence on Sex Differences
EU: Emory University
EPIS: Exploration, preparation, implementation, sustainment
FIC: Fogarty International Center
GBV: Gender based violence
GDP: Gross domestic product
HMU: Hanoi Medical University
(HICs): High-income countries
IDP: Individual development plan
IPRCE: Injury Prevention Research Center at Emory
IPV: Intimate partner violence
LMICs: Low-and-middle income countries
NIH: National Institutes of Health
RE-AIM: Reach, effectiveness, adoption, implementation, maintenance
REDCap: Research electronic data capture system
RCR: Responsible conduct of research
SDGs: Sustainable development goals
TAC: Training advisory committee
TGNC: Trans-gender or gender-non-conforming
US: United States
UMP: University of Medicine and Pharmacy
VAC: Violence against children
